# Forest bathing (*Shinrin-Yoku*) as an integrative strategy for mental and cardiovascular health: a quasi-experimental study in Brazil

**DOI:** 10.3389/fpubh.2026.1817791

**Published:** 2026-05-11

**Authors:** Guilherme Franco Netto, Marco Aurélio Bilibio, Aurélio Matos Andrade, Giovanny Vinícius Araújo de França, Alice Miranda Bentes, Rogério Romeiro Oliveira, Thiago Gabriel Barbosa Ribeiro Cecilio Daher, André Luiz Dutra Fenner, Lorena Covem Rosa Franco Netto, Suzane da Fonseca Durães, Isabella Covem Martins

**Affiliations:** 1 Oswaldo Cruz Foundation, Vice-Presidency of Environment, Care and Health Promotion (VPAAPS), Rio de Janeiro, Brazil; 2 Instituto Brasileiro de Ecopsicologia, Brasília, Brazil; 3 Fiocruz Brasília, Program of Evidence for Health Policy and Technologies (PEPTS), Brasília, Brazil; 4 Ministério da Saúde, Departamento de Ciência e Tecnologia, Secretaria de Ciência, Tecnologia e Inovação em Saúde, Brasília, Brazil; 5 University of Brasília, Brasília, Brazil; 6 Fiocruz Brasília, Health, Environment and Work Promotion Program (PSAT), Brasília, Brazil

**Keywords:** Brazil, cardiovascular disease, forest bathing, mental disorders, nature therapy, quasi-experimental study

## Abstract

Forest bathing (Shinrin-Yoku) therapy has emerged as a promising environmental health intervention with measurable physiological and psychological benefits. Evidence from the United States, Europe, and Asia indicates that exposure to natural environments reduces stress-related biomarkers, including salivary cortisol, anxiety symptoms, and blood pressure. However, Brazilian evidence remains limited, particularly in the Cerrado biome and within the context of the public health system. To analyze the effects of forest bathing therapy, compared with guided urban exposure, on physiological and psychological markers of stress, anxiety, and depression among adults residing in the Federal District, Brazil. A quasi-experimental study was conducted with two parallel groups: an intervention group participating in eight forest bathing sessions and a control group exposed to guided urban environments over the same period. Primary outcomes included systolic and diastolic blood pressure (SBP, DBP), salivary cortisol levels, perceived stress (PSS-10), and psychological symptoms assessed by the Depression, Anxiety and Stress Scale (DASS-21). Intragroup and intergroup comparisons were performed using the Wilcoxon rank-sum test (Mann–Whitney U-test), Stuart–Maxwell test, and Fisher’s exact test, adopting a 5% significance level. Groups were comparable at baseline. No significant intergroup differences were observed for SBP or DBP; however, cortisol reduction was significantly greater in the intervention group (*p* = 0.024). Within-group analyses demonstrated significant reductions in SBP (119.50 → 108.00 mmHg; *p* = 0.001) and salivary cortisol (0.29 → 0.16 μg/dL; *p* = 0.002) among participants exposed to forest bathing. Psychologically, the proportion classified as low stress increased from 17.4 to 52.2% (*p* = 0.006). DASS-21 results showed significant redistribution toward the normal category for anxiety (52.2% → 95.7%; *p* = 0.042) and stress (26.17% → 69.6%; *p* = 0.018). Forest bathing produced acute reductions in physiological and psychological stress markers, supporting its role as a nature-based, complementary public health strategy. These findings highlight its potential integration into Brazil’s Unified Health System (SUS), particularly Integrative and Complementary Health Practices (ICHP), reinforcing the relevance of environmental exposures in mental health promotion.

## 1 Introduction

Forest bathing, a Japanese self-care practice known as *Shinrin-Yoku*, consists of a conscious immersion in the forest environment conducted as a structured and guided activity with a defined purpose and method (1). Unlike general recreational exposure to nature, forest bathing emphasizes intentional engagement through sensory awareness, particularly the deliberate use of the five senses (sight, hearing, smell, touch, and taste) as a central component of the experience. This activity integrates a broader approach to healthcare, recognizing the benefits of contact with nature in prevention and treatment of cardiovascular and mental illnesses, including stress-related disorders, anxiety, and depression (2). Evidence suggests that forest bathing can significantly reduce cortisol levels in the short term, contributing to psychophysiological restoration and stress reduction, promoting physical and mental well-being (3). Incorporating this practice into public health policies has the potential to strengthen the importance of healthy natural environments as determinants of human well-being and quality of life.

According to the World Health Organization (WHO) (4), cardiovascular diseases (CVDs) remain the leading cause of global morbidity and mortality, being responsible for approximately 17.9 million deaths, which corresponds to around 31% of all deaths worldwide. Among these, 85% are due to acute myocardial infarction (AMI) and cerebrovascular accidents (CVAs). It is also estimated that more than 75% of deaths by cardiovascular diseases (CVDs) occur in low and middle-income countries (LMICs), reflecting social inequalities and limitations in access to prevention and treatment (5). In 2021, cardiovascular diseases (CVDs) were the leading cause of deaths in Brazil since the 1960s, accounting for about 28% of deaths (6). In 2023, approximately 390,000 deaths were registered due to cardiovascular causes, mainly due to arterial hypertension, dyslipidemia, smoking, obesity, and sedentary lifestyle (7).

On the other hand, mental disorders represent one of the greatest global public health challenges, affecting, in 2024, approximately 1 billion people worldwide (8). Depression is a leading cause of disability, affecting approximately 5% of the world’s adult population, while anxiety disorders affect about 4% (9). Furthermore, according to the WHO (10), it is estimated that nearly 800,000 people die by suicide each year, making it the fourth leading cause of death among young people aged 15 to 29. In Brazil, it is estimated that 9.3% of the population suffers from anxiety disorders and 5.8% from depression, which are more prevalent than the global average (7). The country also faces high prevalences of suicidal ideation and psychological distress, aggravated by social inequalities, urban violence, and economic insecurity (11).

The report *Connecting Global Priorities: Biodiversity and Human Health* highlights the importance of healthy ecosystems in preventing chronic non-communicable and mental diseases, promoting the One Health concept, which integrates human, animal, and environmental health (12). Similarly, the Convention on Biological Diversity (CBD) and the 2030 Agenda for Sustainable Development recognize the role of natural environments in promoting health and well-being, especially in Sustainable Development Goals (SDGs) 3 (Good Health and Well-being) and 15 (Life on Land) (13, 14).

In countries like Japan and South Korea, national policies have institutionalized forest bathing as a therapeutic and preventive practice, with investments in green infrastructure and certification of therapeutic forests (15). In Brazil, national health policies do not recognize this practice as a health care therapy yet. However, the National Policy on Integrative and Complementary Practices (Política Nacional de Práticas Integrativas e Complementares – PNPIC) of the Unified Health System (SUS), established by Ordinance No. 971/2006 and expanded by Ordinance No. 702/2018, includes therapies based on mind–body practices and contact with nature, such as aromatherapy, meditation, art therapy, and integrative community therapy (ICT), opening space for future incorporations of ecotherapeutic and nature-based health interventions (16, 17). Additionally, the National Health Promotion Policy (NHPP) and the Subsidies for the Construction of the National Environmental Health Policy (NEHP) reinforce the need for integration between environment and health, recognizing the natural environment as a social determinant of health (18, 19).

The central hypothesis of this groundbreaking study in Brazil is that exposure to natural forest environments, through the weekly practice of forest bathing, promotes significant short-term improvements in psychological and physiological indicators when compared to exposure restricted to the urban environment in Brazil’s capital. This hypothesis is justified by growing scientific evidence in Asian and European countries by which the contact with nature can significantly reduce markers of stress, anxiety, and depression through positive modulation of the hypothalamic–pituitary–adrenal axis, reflected in lower salivary cortisol levels and improved cardiovascular regulation (3, 20–22). As a result, this pilot study aims to analyze the effects of forest bathing therapy, compared to strict exposure to the urban environment in the city of Brasília, on biological, physiological, and psychological markers of stress, anxiety, and depression.

## 2 Methods

### 2.1 Study design

This is a quasi-experimental before-and-after (*or pre-post*) pilot study aimed at investigating the effect of interventions in real-world contexts. The research was submitted to ethical review, and it was approved by the Research Ethics Committee of the Oswaldo Cruz Foundation – Fiocruz Rio de Janeiro, CAAE 89025125.9.0000.5248, in accordance with the guidelines and research regulation rules involving human subjects established by Resolution No. 466/2012 of the National Health Council (NHC). Free and Informed Consent Form (TCLE) was signed from all participants, following the ethical principles of research with human subjects, as well as the principles of the Declaration of Helsinki and its subsequent amendments (most recently revised in 2013). This research is a follow-up to the first published article, “Short-term cardiovascular and mental health responses to Shinrin-Yoku (forest bathing): a systematic review and meta-analysis.” (23).

### 2.2 Participants

**Figure 1:**
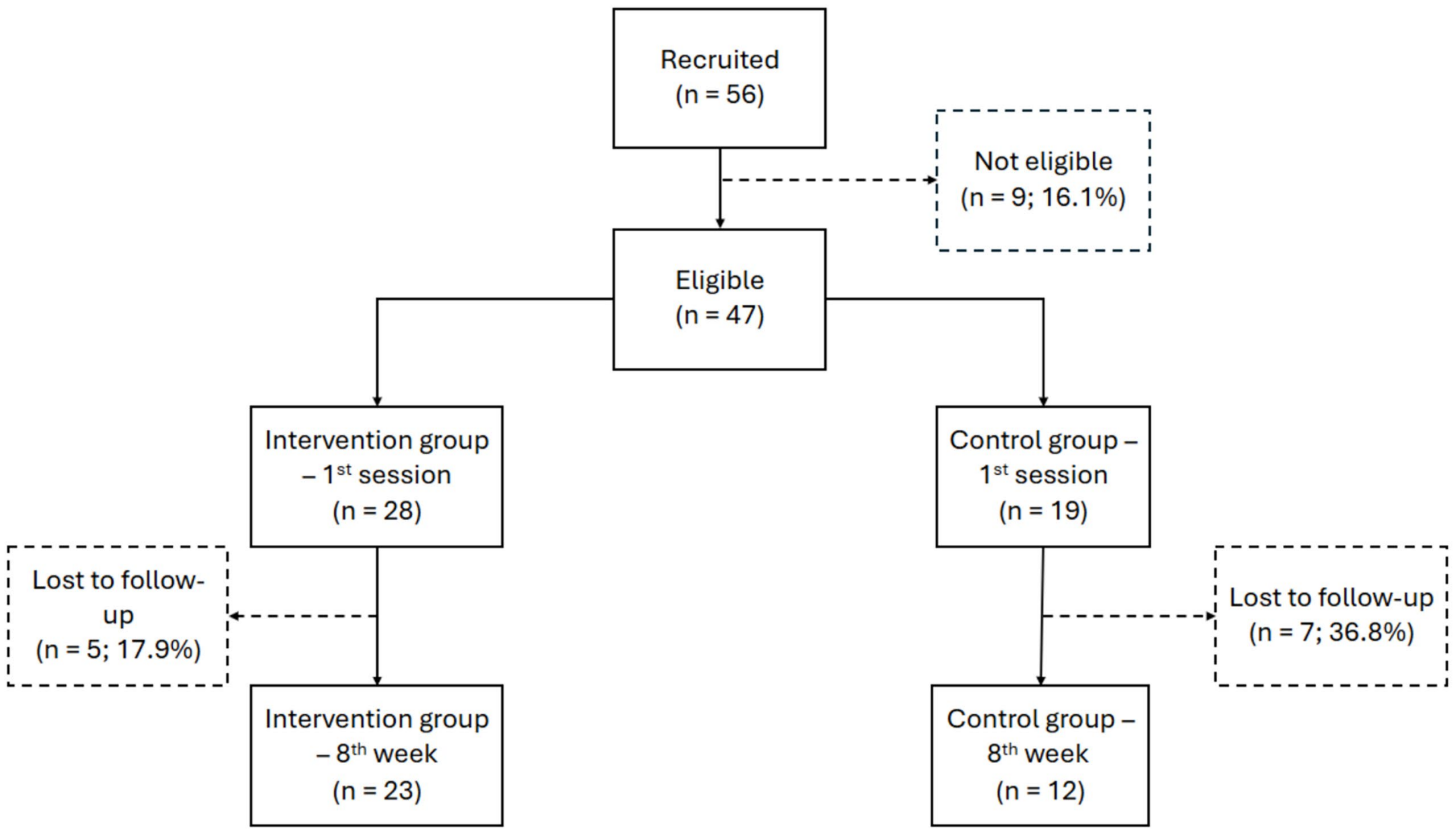
Flowchart of recruitment, eligibility, allocation, and follow-up of study participants. Elaborated by the authors, 2026.

A total of 56 participants of both genders, aged between 18 and 75 years, were recruited for the study (Figure 1). Participants were recruited from the community through social media platforms, and institutional communication channels. Interested individuals were invited to participate on a voluntary basis and contacted the research team for further information and eligibility screening. After the initial screening stage, 9 individuals (16.1%) were considered ineligible due to reported use of psychotropic drugs. In the intervention group (forest bathing), 5 participants (17.9%) lost the follow-up, and only 23 participants completed it. In the control group (urban environment), there were 7 losses to follow-up (36.8%), totaling 12 participants. The allocation into groups occurred in order of registration, starting with the intervention group.

The individuals eligible for the study were healthy individuals or those with a history of mild arterial hypertension (stage 1), with systolic blood pressure between 140 and 159 mmHg and diastolic blood pressure between 90 and 99 mmHg, and/or generalized anxiety disorder associated with restlessness, fatigue, difficulty concentrating, irritability, muscle tension, and sleep disturbances, all residing in Brazil. Participants with mild hypertension were included regardless of antihypertensive medication use; however, medication use was self-reported and monitored to ensure stability during the study period, and no changes in medication were reported during the intervention. The exclusion criteria included individuals with severe heart diseases or using psychotropic medications, which could interfere with the results or compromise the patient’s medication safety during the intervention. These criteria were identified before the intervention through a self-administered questionnaire.

### 2.3 Study locations

The field experiment was conducted between 16 August and 11 October 2025 (late winter to mid-spring) in Brasília, the country’s capital, located in the Federal District, Brazil. In the forest environment, the intervention was developed at *Capivara Trail (Trilha da Capivara)*, in Brasília National Park, at *Mineral Water* (*Água Mineral*), located in the northwest of Federal District, about 6,2 miles from the central area of Brasília. The area is covered by flora typical of the Cerrado biome: vegetation composed of medium-sized trees with thick and twisted branches and trunks, 15 to 25 m high, which is the result of a long evolutionary process in which the plants sought to adapt to difficult environmental conditions, such as water stress, lack of air humidity, and soil acidity (24, 25). The vegetation in the study area includes a mix of gallery forest species, with higher density of taller trees near water bodies, interspersed with shrubs and understory vegetation, typical of riparian Cerrado formations. The soil is predominantly well-drained, acidic, and nutrient-poor, characteristic of the Cerrado biome, with a layer of leaf litter contributing to moisture retention (26).

**Figure 2:**
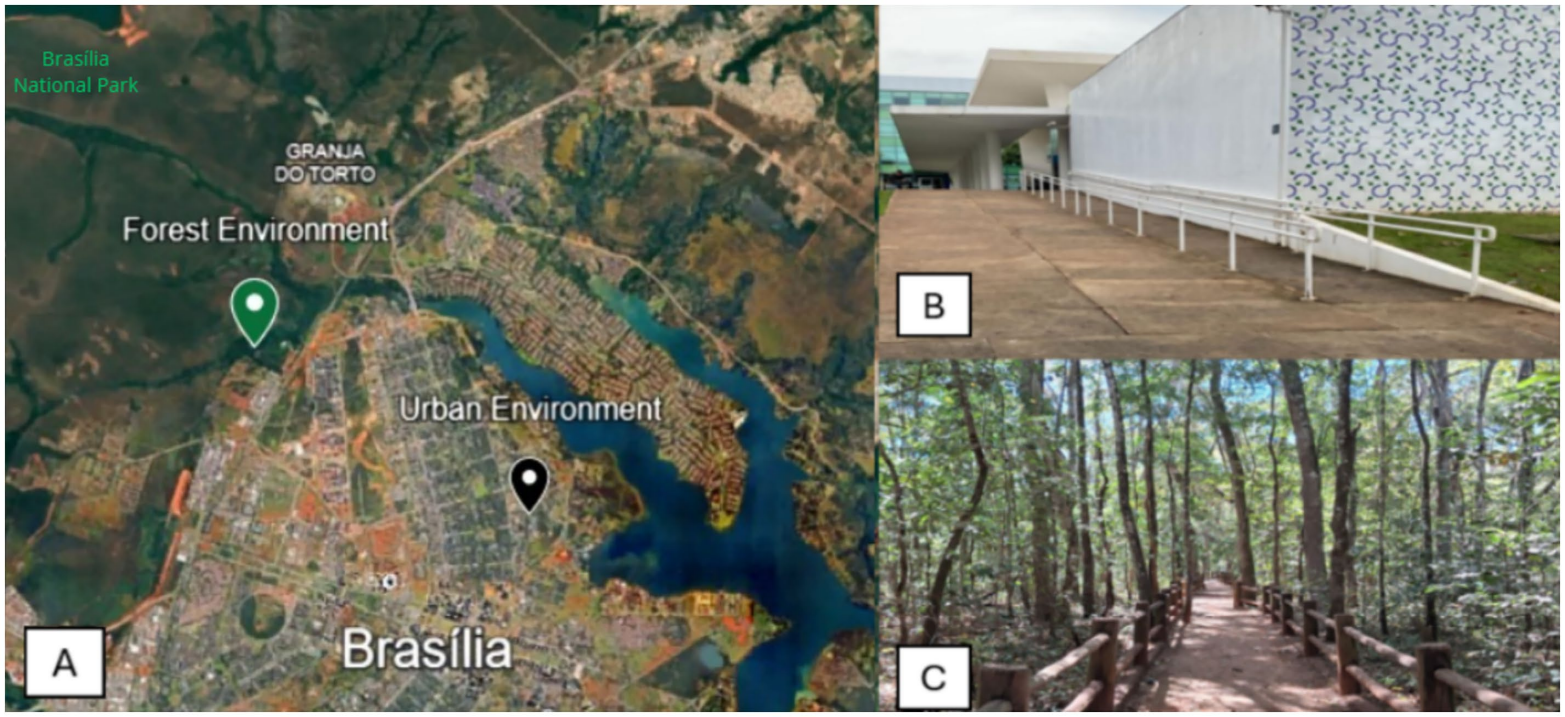
Map showing the geographic location of urban and forest environments **(A)** and *in situ* photos of the urban (map from *Google Earth*, map data: Google, Airbus, Vexcel Imaging, US, Inc., 2026) **(B)** and forest **(C)** environments, Brasília, Brazil, 2025.

The *Capivara Trail* is in a Cerrado gallery forest area, with the presence of a water spring and a catchment basin associated with this spring, forming a small lake adjacent to the trail. The complete route has a total length of approximately 0.7 miles; however, for the purposes of this research, only half of the trail was used, with a length of about 0.3 miles (Figure 2). The trail is flat for most of its length with wooden side markers. Considering that the study period encompassed the transition from late winter to mid-spring, slight natural changes in the landscape were observed, including gradual increases in vegetation density, leaf renewal, and subtle variations in color and humidity. During this period, relative humidity levels were generally low, ranging approximately between 30 and 50%, consistent with the dry season of the tropical savanna climate (Aw) (27). These conditions contributed to drier soil surfaces and lower water levels in smaller streams. Despite these variations, shaded areas and proximity to water sources provided a relatively stable and comfortable sensory environment for the participants.

The urban environment was selected as the control location. The experiment was conducted in a room, in a building located in the northern region of Brasília, with a view from the building extending to the urban landscape with some wooded areas. The room’s air conditioning was off during the experiment (Figure 2).

Meteorological data were collected from the National Institute of Meteorology (Instituto Nacional de Meteorologia, INMET) (location: 15°38'28" south latitude and 48°1'15" west longitude). The average temperature during the study period was 21.6 °C to 24 °C, the weather was sunny and without precipitation on the days of the experiments (28). According to the Köppen-Geiger climate classification, the climate observed in the Federal District is tropical savanna (Aw), characterized by a tropical climate with a well-defined dry season (29).

### 2.4 Experimental design

The forest bathing experiment was conducted in sessions over 8 weeks. The measurement of the outcomes evaluated in this article occurred in the first session (baseline) and in the eighth session in both groups. Before the cortisol measurement, participants were instructed to avoid alcohol, smoking and eating for at least 1 h prior to sample collection, and to remain seated and relaxed for 5 min before the saliva sample was taken. It is noteworthy that only the intervention group held weekly meetings throughout the project period. Therefore, the control group met only twice, for the initial and final data collection, in weeks corresponding to the intervention group’s schedule. The meeting point for the intervention group was a tent at the beginning of *Capivara Trail* in Brasília National Park, while for the control group, the meetings took place in a building in the northern region of Brasília. Before starting the experiment, the objectives of this pilot study and the experimental protocol were explained, and general instructions were given to the participants regarding access, care, and food for forest bathing. Saliva samples from sessions 1 and 8 were collected separately and analyzed in batches to avoid cross-contamination and ensure accurate temporal comparison of cortisol levels. After this orientation, the participants were divided into intervention and control groups according to their registration order, and the experiment in the forest area took place on different days than in the urban area.

On the days of the experiment, participants from both groups arrived at the experiment locations between 8:00 and 9:00 a.m. After arriving at the experimental/control location, the participants rested in a chair for 5 min and then began data collection: blood pressure (BP), saliva sample for cortisol measurement, and the scales, with an average duration of 25 min for each participant. Blood pressure and saliva samples were collected before and after the experiment in the intervention group and only once in the control group at the corresponding time points (baseline and final session).

In the intervention group, the participants were instructed by trained guides who applied the Forest Bathing weekly protocol. The walk was relatively slow, with the group maintaining the same pace, and there was one guide in front and another behind to coordinate the rhythm. At the beginning of the practice, the participants were informed that forest bathing, although performed in a group, constitutes an individual and silent experience. At this time, they were also instructed to turn off their electronic devices.

The average duration in the intervention group was between 2 and 2.5 h, structured in five stages. The five-stage procedure was developed by the research team with modifications to optimize sensory engagement and participant safety (15). The same structured activities were performed each week, maintaining consistency across the 8-week intervention.

The first stage, called *the Connection Portal*, consisted of a guided concentration meditation in an upright position, lasting approximately 10 min, during which participants were instructed to focus on breathing and body awareness. The second stage, the *Portal of the Senses*, was a meditation aimed at expanding attention and sensitivity to the forest’s sensory stimuli – touch, sight, smell, and hearing – with an average duration of 15 min. Participants were guided to deliberately observe and notice environmental details, such as textures, colors, sounds, and scents, enhancing sensory immersion.

The third stage, called the *Portal of Encounters*, was the longest of the practice, lasting approximately 1 h. During this phase, participants walked the trail individually and silently, seeking to establish meaningful encounters and interactions with the natural elements of the environment, as well as choosing a place to stay. The fourth stage, called the *Portal of Quietness*, was when the volunteers remained seated or lying down in their previously chosen locations, with an average duration of 15 min.

Finally, the fifth stage, called the *Tea Ceremony*, corresponded to the closing moment of the practice, in which the Forest Bathing guides organized and served tea to the participants, who were invited to share their perceptions, experiences, and learnings from the experience with the group, lasting approximately 30 min.

For the control group, participants spent an equivalent duration (approximately 40 min) in the urban environment, seated or performing light walking within the room and adjacent urban area.

### 2.5 Selection of health measurement instruments

The research protocol was developed based on a systematic literature review of methods for measuring cardiovascular and mental health in studies on forest bathing (20–22, 30). For cardiovascular health, selected physiological markers were systolic and diastolic blood pressure (BP), measured using the Omron HEM-6181 device.

As a biological marker of stress and anxiety in participants, salivary cortisol levels were measured before and after the intervention using the Salivette system. Each participant held two pieces of absorbent cotton in their mouth for 2 min, and the saliva contained in these cotton pads was subsequently extracted by centrifugation. The samples were immediately frozen and transported to the Larc, Inc. laboratories (Brasília, Brazil). Each sample consisted of a 0.8 mL aliquot of saliva, and its cortisol concentration was analyzed by electrochemiluminescence.

In addition, two assessment instruments were used as psychological or behavioral markers: the Perceived Stress Scale (PSS-10) (31) and the Depression, Anxiety and Stress Scale (DASS-21) (32). Both have validated Portuguese versions and are easy to complete, taking an average of 15 min.

The PSS-10 assesses the extent to which individuals perceive life situations as stressful. Developed by Cohen et al. (33) and adapted for Brazilian context by Luft et al. (29), PSS-10 consists of 10 self-applicable items that explore perceptions of unpredictability, lack of control and overload. Each item is scored on a Likert scale from 0 (never) to 4 (always), and the total sum provides a measure of perceived stress, with higher scores indicating greater perception of stress.

The DASS-21 evaluates depression, anxiety, and stress scale. An abbreviated and adapted Brazilian (30) version of Lovibond and Lovibond (34) was used, containing 21 items, equally distributed among 3 subscales. Each item is scored on a Likert scale from 0 (not applied at all) to 3 (applied much of the time, or most of the time), reflecting the frequency or severity of negative emotional experiences.

The collection of BP and salivary cortisol was conducted under the supervision of a nursing professional, while the administration of the PSS-10 and DASS-21 scales was accompanied by a psychology professional. During the research planning phase, 6 specialists of different specialties were consulted: psychology, nursing, epidemiology, public health, public policies and environment.

Initially, the description of the investigated population was carried out based on the sociodemographic, clinical, behavioral, and psychological variables obtained in the study, through absolute and relative frequencies. For analytical purposes, the participants were classified according to gender (man or woman) and level of education, grouped in complete high school, university education in progress, undergraduate or graduate. The variable race/color was defined according to self-reference, being categorized as white, black or brown (35). The socioeconomic status was estimated from the monthly family income, expressed in number of minimum wages, and subsequently organized into 2 categories: up to 5 minimum wages or 5 minimum wages or more. The nutritional status was established based on body mass index (BMI) and classified as low weight (<18,5 kg/m^2^), eutrophy (18,5 to 24,9 kg/m^2^), overweight (25,0 a 29,9 kg/m^2^) or obesity (≥30 kg/m^2^) (36). In addition, participants’ physical activity was registered based on self-report, being categorized as yes or no. In the psychometric domain, the PSS-10 score was categorized as low (1–13), moderate (14–26) or high (27–40). Furthermore, the DASS-21 scores were converted to interpretative categories corresponding to the 3 domains evaluated and classified as: normal (0 to 9), mild (10–13), moderate (14–20), severe (21–27), or extremely severe (28 or more) (38).

### 2.6 Data extraction and analysis

The data collected through the questionnaires, as well as the variables measured during the study, were initially typed and structured in an electronic spreadsheet using the software Microsoft Excel®. This stage involved manual review and preliminary conference of the information to ensure standardization and completeness. After this initial organization, the database was exported and converted to the format compatible with Stata, version 16.1 (StataCorp LLC) for statistical analysis. An exploratory analysis was conducted to identify any inconsistencies, missing values, *outliers,* and possible typing errors, ensuring the quality and reliability of the database used in subsequent analyses.

The comparison of characteristics between control and intervention groups included sociodemographic, clinical, behavioral, and categorical psychological variables. For each category, absolute and relative frequencies were calculated, and the differences between groups were evaluated by Fisher’s exact test. For continuous variables, data distribution was examined using the Shapiro–Wilk normality test, considering *p* < 0.05 as indicative of deviation from normality.

The comparison of basal continuous variables between control and intervention groups was performed using the Wilcoxon rank-sum test (Mann–Whitney), as part of the variables revealed non-normal distribution. Measures of central tendency and dispersion were calculated, including mean, standard deviation, median, and interquartile intervals (Q1 and Q3).

The effect of the intervention on the hemodynamic and biochemical variables was evaluated based on the differences between post-intervention and pre-intervention values, which were compared between control and intervention groups using the Wilcoxon rank-sum test. The differences between PSS-10 and DASS-21 scores, before and after the intervention, in control and intervention groups were also analyzed using this test. Additionally, for the intervention group, pre- and post-intervention values of the variables SPB, DBP and cortisol were compared using the paired Wilcoxon test.

Changes in the categories of PSS-10 and DASS-21 between the pre- and post-intervention moments were examined in the intervention group using the Stuart-Maxwell test for marginal homogeneity. For both scales, alluvial type graphs were used to visually represent transitions between categories over time.

Hypothesis tests were interpreted by adopting a significance level of 5%. Graphical visualizations for data synthesis and exploration were generated by R Studio software version 4.3.0.

## 3 Results

The sociodemographic and clinical profile of the participants was relatively homogeneous between the control and intervention groups, with no statistically significant differences for the variables sex, education, race/color, family income and nutritional status (p ranging from 0.237 to 0.737). The sample was mainly composed of women (68.6%), people with high education, especially graduate (65.7%) and predominantly white (60.0%). Family income did not differ between groups, with highlight in 66.7 and 56.5% for income above three minimum wages in the control and intervention groups, respectively, while the nutritional status presented identical proportions of eutrophy and overweight (both 45.7%). The occupation was diversified, more often “Others/Not specified” (*n* = 18) (Table 1).

**Table 1:** Comparison of characteristics between control and experimental groups (*n* = 35).

Characteristics	Total		Control		Intervention		*p*-value*
	(*n* = 35)		(*n* = 12)		(*n* = 23)		
	*n*	%	*n*	%	*n*	%	
Gender							0.576
Man	11	31.4	4	33.3	7	30.4	
Woman	24	68.6	8	66.7	16	69.6	
Educational level*							0.737
High school/technical education	2	5.7	1	8.3	1	4.4	
Incomplete undergraduate education	2	5.7	0	-	2	8.7	
Complete undergraduate education	8	22.9	2	16.7	6	26.1	
Complete graduate education	23	65.7	9	75.0	14	60.9	
Race/color							0.237
White	21	60.0	5	41.7	16	69.6	
Black	2	5.7	1	8.3	1	4.4	
Brown	12	34.3	6	50.0	6	26.1	
Income (minimum wages)							0.721
Until 3	14	40.0	4	33.3	10	43.5	
3 or more	21	60.0	8	66.7	13	56.5	
Nutritional status**(n = 1)							0.294
Eutrophy	16	45.7	4	33.3	12	52.2	
Overweight	16	45.7	8	66.7	8	34.8	
Obesity	2	5.7	0	-	2	8.7	
Religion							0.178
Catholic	7	20.0	4	33.3	3	13.0	
Evangelical	2	5.7	1	8.3	1	4.4	
Spiritist/spiritualist	3	8.6	0	-	3	13.0	
Afro-Brazilian origin	1	2.9	1	8.3	0	-	
No religion/others	22	62.9	6	50.0	16	69.6	
Occupation							0.378
Health	7	20.0	2	16.7	5	21.7	
Biological sciences	1	2.9	0	-	1	4.4	
Human sciences	3	8.6	1	8.3	2	8.7	
Applied social sciences	5	14.3	1	8.3	4	17.4	
Engineering and exact sciences	1	2.9	1	8.3	0	-	
Others/not specified	18	51.4	7	58.3	11	47.8	
Practice of physical activities							0.685
No	9	25.7	4	33.3	5	21.7	
Yes	26	74.3	8	66.7	18	78.3	
Perceived Stress Scale (PSS-10)							0.138
Low	9	25.7	5	41.7	4	17.4	
Moderate	22	62.9	7	58.3	15	65.2	
High	4	11.4	0	-	4	17.4	
DASS-21 Depression							0.877
Normal	24	68.6	10	83.3	14	60.9	
Mild	6	17.1	1	8.3	5	21.7	
Moderate	3	8.6	1	8.3	2	8.7	
Severe	1	2.9	0	-	1	4.4	
Extremely Severe	1	2.9	0	-	1	4.4	
DASS-21 Anxiety							0.800
Normal	21	60.0	9	75.0	12	52.2	
Mild	6	17.1	2	16.7	4	17.4	
Moderate	5	14.3	1	8.3	4	17.4	
Severe	2	5.7	0	-	2	8.7	
Extremely severe	1	2.9	0	-	1	4.4	
DASS-21 Stress							1.000
Normal	9	25.7	3	25.0	6	26.1	
Mild	7	20.0	3	25.0	4	17.4	
Moderate	12	34.3	4	33.3	8	34.8	
Severe	3	8.6	1	8.3	2	8.7	
Extremely severe	4	11.4	1	8.3	3	13.0	

* Fisher’s exact test. ** Number of missing data per variable. Elaborated by the authors, 2026.

There was a high prevalence of self-reported lifestyle-related physical activity among the participants (74.3%). The comparative analysis between groups showed no significant differences in physical activity practice (*p* = 0. 685) or nutritional status (*p* = 0. 294). Mental health indicators showed a predominance of moderate stress (62.9%) on the PSS-10, while the DASS-21 indicated a higher proportion of participants in the normal category for depression and anxiety, with no significant differences between groups (*p* ranging from 0.800 to 1.000) (Table 1).

At the beginning of the study, no statistically significant differences were observed between the groups for any of the variables evaluated. The mean age was similar between the groups (47.50 ± 11.0 years in the control group vs. 51.65 ± 13.74 in the intervention group), as were weight (69.03 ± 10.36 kg vs. 69.47 ± 12.33 kg), height (165.67 ± 7.58 cm vs. 167.74 ± 7.05 cm) and BMI (25.12 ± 3.19 kg/m^2^ vs. 24.3 ± 22.77 kg/m^2^) (Table 2).

**Table 2:** Comparison of variables between control and intervention groups.

Variables	Control						Intervention						*p*-value*
	*n*	Mean	SD	Median	Q1	Q3	*n*	Mean	SD	Median	Q1	Q3	
Age (years)	12	47.50	11.00	46.00	39.00	59.50	23	51.65	13.74	56.00	41.00	60.00	0.375
Weight (kg)	12	69.03	10.36	71.25	59.90	77.00	22	69.47	12.33	67.50	61.00	81.00	0.928
Height (cm)	12	165.67	7.58	166.00	161.00	168.00	23	167.74	7.05	167.00	163.00	175.00	0.542
BMI (kg/m^2^)	12	25.12	3.19	26.50	21.76	27.12	22	24.68	3.32	24.33	22.77	26.53	0.471
SBP (mmHg)	12	115.33	12.08	114.50	108.00	125.50	20	125.70	19.11	119.50	112.00	131.50	0.199
DBP (mmHg)	12	75.25	12.61	77.00	63.50	84.00	20	73.20	8.02	74.00	69.50	77.50	0.533
Cortisol (μg/dL)	12	0.22	0.10	0.22	0.15	0.30	22	0.31	0.10	0.29	0.24	0.37	0.063

* Two-sample Wilcoxon rank-sum test (Mann–Whitney). Elaborated by the authors, 2026.

**Figure 3:**
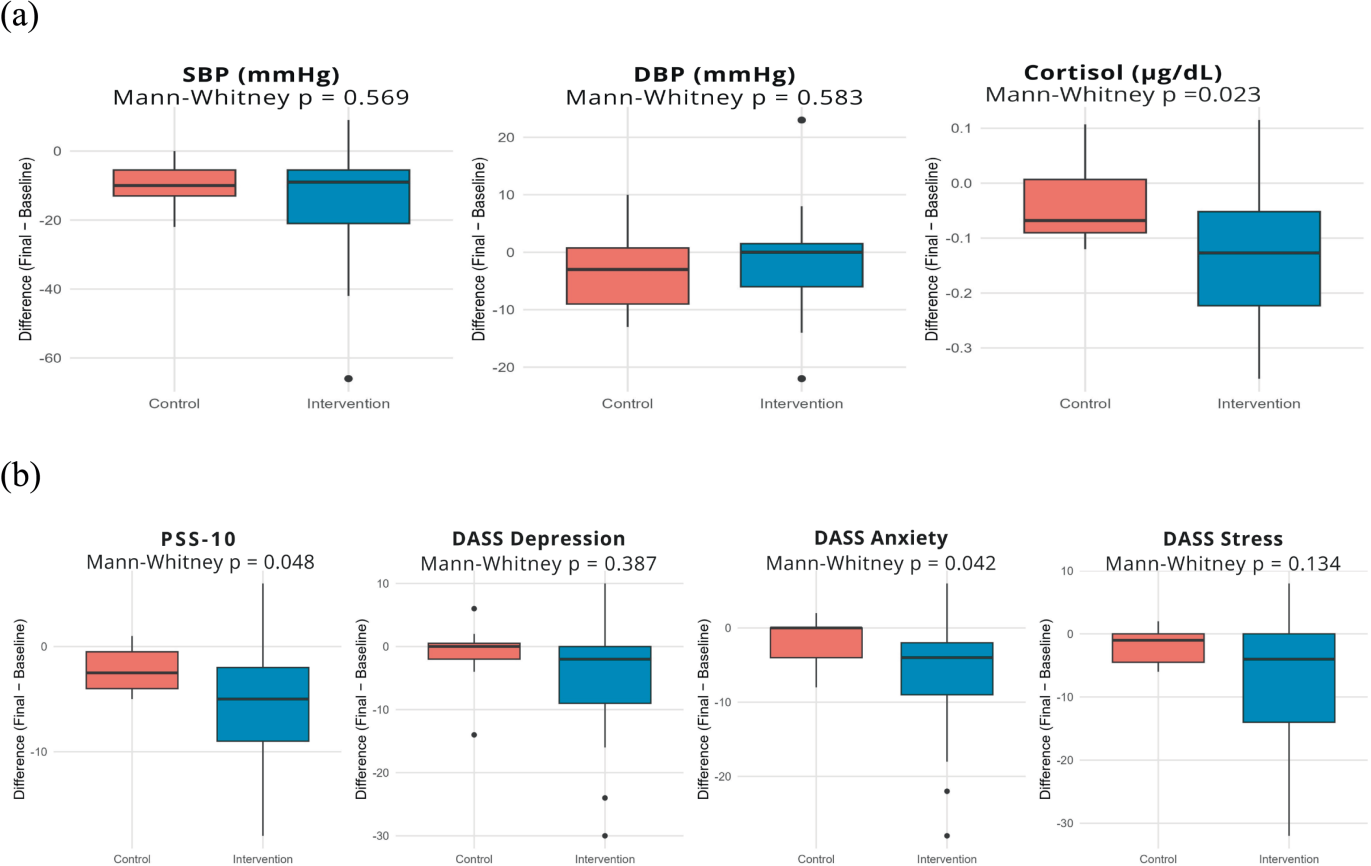
Boxplots of the differences between pre (1^st^ session) and post-intervention (8^th^ session) for **(A)** hemodynamic and biochemical variables and **(B)** PSS-10 and DASS-21 scales comparing control and intervention groups. Elaborated by the authors, 2026.

When comparing the differences between the first and eighth sessions in the intervention and control groups, it was observed that forest bathing did not produce statistically significant differences in the BP, as no differences were identified in the median of SBP (−10.0 mmHg in the control group and −9.0 mmHg in the intervention; *p* = 0.569) and DBP (−3.0 mmHg in the control and 0.0 mmHg in the intervention; *p* = 0.583) between groups. In contrast, salivary cortisol showed a significantly greater reduction among participants exposed to forest bathing (−0.13 μg/dL), compared with the control group (−0.07 μg/dL), reaching statistical significance (*p* = 0.024) (Figure 3A; Supplementary Table 1S).

Additionally, when analyzing the differences between the first and eighth sessions in psychometric outcomes, a greater reduction of perceived stress scores (PSS-10) was observed in the intervention group (median = −5.0) compared to the control group (median = −2.5), a statistically significant difference (*p* = 0.048). A similar result was observed for anxiety assessed by the DASS-21, with a more pronounced reduction in the intervention group (median = −4.0) compared to the control group (median = 0.0), also a statistically significant difference (*p* = 0.042). On the other hand, although the intervention group presented showed a tendency toward greater reductions in depression scores (median = −2.0 vs. 0.0) and DASS-21 stress scores (median = −4.0 vs. −1.0), when compared to the control group, these differences did not reach statistical significance (*p* = 0.387 and *p* = 0.134, respectively) (Figure 3B; Supplementary Table 3S).

**Figure 4:**
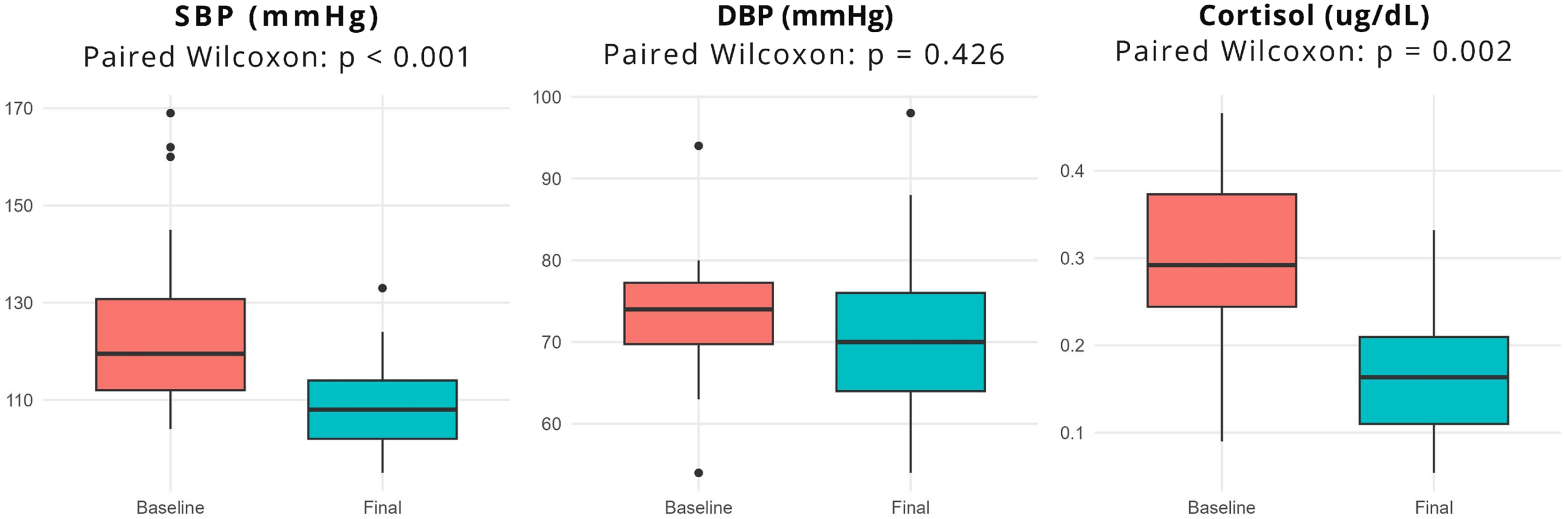
Boxplots of pre- and post-intervention distributions of hemodynamic and biochemical variables in the intervention group. Elaborated by the authors, 2026.

In the intragroup analysis, considering only the intervention group (before and after), the forest bathing evidenced significant physiological changes, highlighting the expressive reduction of SBP, with the median decreasing from 119.50 mmHg to 108.00 mmHg (*p* < 0.001), which indicates beneficial effect of forest bathing on cardiovascular regulation. Although DBP showed a reduction in the median from 74.00 to 70.00 mmHg (*p* = 0.426), this variation was not statistically significant. In contrast, salivary cortisol levels showed a significant reduction, with the median decreasing from 0.29 μg/dL to 0.16 μg/dL (*p* = 0.002) (Figure 4; Supplementary Table 2S).

**Figure 5:**
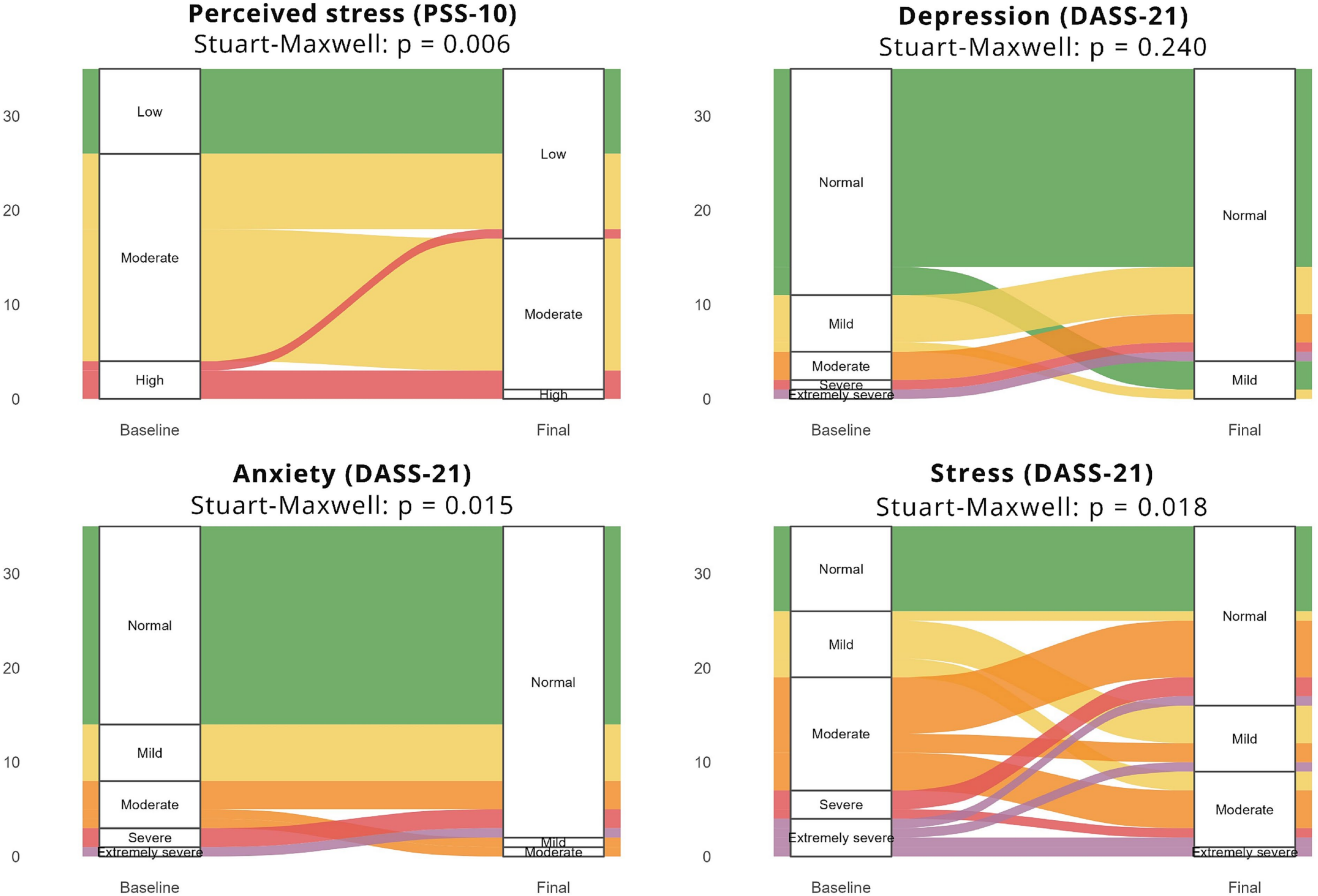
Alluvial graphs representing transitions of participants between the categories of PSS-10 and DASS-21, before and after the intervention. Elaborated by the authors, 2026.

The intervention generated a statistically significant reduction in perceived stress, leading to a significant change in the distribution of PSS-10 categories. Before the intervention, most participants presented moderate stress (65.2%), followed by high or low stress categories, with four participants in each category (18.2%). After intervention, there was a marked increase in individuals with low stress, which represented half of the sample (52.2%), accompanied by a decrease in the moderate (43.5%) and high (4.4%) categories (*p* = 0.006) (Figure 5).

The categories of DASS-21 revealed different patterns of change after intervention. For depression, there was an increase in the proportion of participants classified as “normal” (from 60.9 to 82.6%); however, this variation did not reach statistical significance in pre- and post-intervention distributions (*p* = 0.240). A significant change was observed for anxiety levels: the proportion of participants classified as “normal” almost doubled, from 52.2 to 95.7%, accompanied by a reduction in the *mild, moderate, severe and extremely severe* categories; this redistribution was statistically significant (*p* = 0.015). In the research of stress levels assessed by the DASS-21, the “normal” category almost tripled, increasing from 26.1 to 69.6%, due to significant migration from the other categories (*p* = 0.018) (Figure 5).

In addition, the analysis of individual transitions between categories showed that the reduction of stress and anxiety occurred predominantly by progressive migration from more severe categories to milder levels, especially from moderate and high on PSS-10, and from mild and moderate grades to normal on the DASS-21. There is also the complete disappearance of severe and extremely severe categories for depression, anxiety, and stress in the post-intervention, reinforcing the beneficial effect of intervention on reducing the severity of psychological symptoms (Figure 5).

## 4 Discussion

The results of this pilot study suggest that the regular practice of forest bathing over 8 weeks produced statistically significant physiological effects, with a reduction in systolic blood pressure and decreased levels of salivary cortisol in the intervention group. These findings indicate that systematic exposure to natural environments may contribute to the modulation of physiological mechanisms related to stress, which is consistent with the study’s initial hypothesis and reinforces a growing aggregation of international evidence that points to benefits associated with nature-based interventions (23, 39–41). Comparing the differences of estimates before and after the intervention of the second group, it was identified a greater reduction in cortisol levels among participants exposed to the forest environment, when compared to the control group, suggesting possible additional benefits from the intervention.

The homogeneous sociodemographic profile among groups, without statistically significant differences, reinforces the internal validity of the findings by reducing confusion biases. The predominance of women and people with a graduate degree corroborates the findings of Antonelli et al. (3) that revealed greater female adherence to nature interventions, observing greater sensitivity to stress reductions (−18% in PSS-10; *p* < 0.01) in a sample composed mostly by women (62%). Regarding socioeconomic level, the predominance of participants with undergraduate or graduate education in this study may limit the generalizability of the results, as populations with lower educational attainment tend to respond differently to health promotion programs. Compared to the study of Kobayashi et al. (42) restricted to young men (20–29 years) from Japanese universities, this study revealed more significant reductions in cortisol levels in the intervention group (−0.29 μg/dL before vs. −0.16 μg/dL after the intervention). However, it is important to note that the post-intervention cortisol value of 0.16 μg/dL in the intervention group is at the lower end of the normal physiological range, which may indicate very low hypothalamic–pituitary–adrenal (HPA) axis activation or even transient exhaustion. This contrasts with the pre-intervention value of 0.29 μg/dL and with reported values in the study by Hunter et al. (43), where salivary cortisol levels around 0.3 μg/dL were observed for morning measurements. The relatively small absolute difference between pre- and post-intervention cortisol levels suggests that cortisol changes alone may not fully account for the observed reduction in PSS-10 scores.

In a systematic review and meta-analysis carried out by Shim et al. (44) it was found that factors such as age, number of participants, study design, female participation rate and BMI were significantly associated with therapeutic effects related to forest environments. The high prevalence of physical activity (74.3%) in the sample also provides some consideration, as active individuals tend to have more efficient autonomic regulation and lower cardiovascular reactivity. However, Song et al. (45) reported that forest bathing produces additional effects even in physically active people, with increased parasympathetic tone in recreational athletes (↑RMSSD in 12%; *p* = 0.03). Furthermore, this study revealed a significant intragroup reduction in systolic blood pressure in the intervention group (−8.5 mmHg; *p* = 0.001) which reinforces the hypothesis of incremental benefits regardless of baseline physical activity levels.

The absence of differences between control and intervention groups for SBP, DBP and cortisol contrasts with findings from meta-analyses that reported clear differences between exposed and not exposed. In the systematic review of Shim et al. (44), considering the combined effect of the included studies, there was a reduction in standardized average differences only for SBP (−0.932; 95% CI: −1.690 to −0.175), comparing the exposed and not exposed groups. There was no observed association with reduction of DBP (−0.434; 95% CI: −0.901 to 0.033) and cortisol concentrations (−1.023; 95% CI: −2.105 to 0.059). High heterogeneity was observed when the combined effect of the studies was analyzed (SBP: I^2^ = 91%; DBP: I^2^ = 81%; Cortisol: I^2^ = 90%), which increases the uncertainty as to the real size of the effect, requiring caution in the interpretation and extrapolation of the overall estimate.

The intragroup analysis, restricted to the intervention group, showed significant reduction of median SBP (119.50 → 108.00 mmHg; *p* = 0.001) and cortisol (0.29 → 0.16 μg/dL; *p* = 0.002). These values are partially comparable to those reported by Li et al. (46), whose participants reduced SBP by −7.5 mmHg (p = 0.002) and cortisol by −15% (*p* < 0.01), although without reporting absolute cortisol concentrations. In addition, it is consistent with the study of Hunter et al. (43) in adults residents of urban areas, in which experiences in natural environments resulted in an additional reduction of 21.3% per hour in salivary cortisol levels, in addition to the expected 11.7%, being the highest efficiency observed in exposures between 20 and 30 min. A study with hypertensive patients conducted by Zhang et al. (47) revealed a reduction of −12.3 mmHg in SBP (*p* < 0.001), higher than that observed in healthy populations. It helps to explain the effects between groups in our study, whose sample had a mean BMI of 24.7 kg/m2 and low chronic illness. Thus, the intragroup effects appear to be consistent with the profile of a healthy population but reinforce the need to test the protocol in groups of clinical hypertensive patients of grade 2 and 3, as well as only in the older adult population.

Regarding the perceived stress, the intervention promoted a consistent redistribution of PSS-10 categories, with an expressive increase in the proportion of participants classified as low stress and a concomitant reduction of moderate and high stress categories after the intervention. A study conducted in Thailand (35) used the PSS-10 to categorize the stress levels of 94 volunteer participants, identifying that most had moderate stress (78; 83.0%). Meanwhile, a study conducted in Tuscany, Italy (48), with 29 healthy participants, demonstrated correlation between the PSS-10 and electrodermal activity (EDA) (R = 0.25; *p* < 0.05). Specifically, the comparison of EDA before and after exposure showed a reduction of 6.96 μSiemens (95% CI: 11.262 to 2.65). These results indicate that EDA is a sensitive marker for evaluation of changes in the activation of the sympathetic nervous system under stress conditions. Both studies did not analyze the impact of intervention on PSS-10 results. However, a US study (49) showed the reduction of perceived stress score after walking in a forest environment by 1.4 points (95% CI: 2.6 to 0.2). Thus, the stress reduction evaluated by PSS-10 can also be observed in other outdoors intervention formats.

The significant improvement in the domains of DASS-21 before and after the intervention, especially anxiety (“normal” increased from 52.2 to 95.7%; *p* = 0.015), converges with the findings of the clinical trial conducted by Bielinis et al. (50) which reported a reduction of around 22% in anxiety scores (*p* < 0.001) after *Shinrin-Yoku* sessions. However, Paredes-Céspedes et al. (51) warn that these effects are more pronounced in the short term, with a return to baseline levels after 6 to 12 weeks, highlighting the need for longitudinal studies.

From a public health perspective, these types of intervention dialogue with contemporary approaches to health promotion, recognizing the role of the environment as a social and ecological determinant of health. Meinköhn et al. (52) demonstrated that continuous nature immersion programs led 10 to 18% reduction in visits for mild psychosomatic complaints, with estimated annual savings of US$ 70 to US$ 150 per year/user. In the Brazilian context, forest bathing should be considered within the framework of existing health policies, such as the National Health Promotion Policy (NHPP – Ordinance GM/MS No 2.446/2014) and the PNPIC, which already recognizes non-biomedical care strategies aimed at reducing stress, strengthening the link with territory and promotion of well-being. Thereby, this study contributes to providing primary empirical data in a context still incipient in the country, opening space for future discussions on feasibility, cost-effectiveness, social acceptance, and applicability in the public health system.

Furthermore, the analysis of individual transitions between categories showed that reduction of stress and anxiety occurred predominantly by progressive migration from more severe categories to milder levels, especially from moderate and high PSS-10, and from mild and moderate grades to normal grade on the DASS-21. There is also the complete disappearance of severe and extremely severe categories for depression, anxiety and stress in the post-intervention, reinforcing the beneficial effect of the intervention on reducing the severity of psychological symptoms (Figure 5).

### 4.1 Limitations of this study

The interpretation of the findings of this pilot study should consider its limitations. The lack of statistical significance in some comparisons may be related to methodological characteristics of the study, especially the small sample size and the asymmetry between groups due to losses during follow-up. A relatively high drop-out rate was observed in both groups, particularly in the control group, which may have introduced selection bias and reduced the comparability between groups. Although no formal secondary analysis of drop-out reasons was conducted, informal reports from participants and field observations suggest that factors such as scheduling constraints, lack of perceived engagement in the urban control condition, and competing personal or professional demands may have contributed to withdrawal.

The higher drop-out rate in the control group, compared to the intervention group, may be partially explained by lower motivation and reduced experiential engagement, as the control condition did not involve immersive or restorative environmental exposure. This difference highlights the potential influence of intervention attractiveness and participant expectations on adherence in nature-based studies. Such limitations may have reduced the statistical power needed to detect intergroup differences, especially in psychosocial outcomes, as evaluated by the PSS-10 and DASS-21. Additionally, no stratified randomization was performed, which may have resulted in slight imbalances between groups regarding participants with stage 1 arterial hypertension, potentially influencing baseline comparability and intervention effects.

It is important to note that, although the control condition did not include immersive forest exposure, participants spent an equivalent duration (approximately 2 to 2.5 h) engaged in structured activities designed to standardize mental load, fatigue, and attentional engagement. However, from a methodological perspective, while efforts were made to ensure comparability in structural aspects between groups, differences in social-ritual components, as the Tea Ceremony, remain potential confounding factors.

In addition, the highly educated and mostly female profile limits the generalization of the findings to more diverse populations in socioeconomic and cultural terms. Another point to consider is that uncontrolled factors, such as variability in sleep, weekly workload and external stressful events, may have influenced both psychological and physiological markers. Finally, because it is a study with intervention in the natural environment, climatic conditions and variable social interactions may have acted as confounding factors.

### 4.2 Public health, equity and policy implications

In the field of Public Health, the incorporation of interventions based on nature as structuring strategies for health promotion should be understood not only as individual care practices, but as actions inserted into broader territorial, social and environmental contexts, in line with the principles of health promotion and comprehensive care.

Populations experiencing greater social vulnerability often live in areas with lower vegetation cover and higher exposure to adverse environmental factors, limiting equitable access to nature-based practices. Thus, the promotion of forest bathing should be considered to promote equity, articulating public policies on urban planning, environmental preservation, and the guarantee of territorial rights.

Another relevant element refers to the risk of individualization of care if the practice is implemented in a disarticulated way of public policies and collective actions. Although the individual benefits are consistent, the Health Promotion approach, as recommended by the National Health Promotion Policy (PNPS), emphasizes interventions capable of acting on life contexts and structural determinants of health. Thus, forest bathing holds greater potential when incorporated into collective strategies, territorialized and integrated into local care networks, while avoiding commodification, elitism, or exclusivity.

From the perspective of health system organization, nature-based interventions have potential cost-effectiveness, especially in the field of mental health and prevention of stress-related diseases. The forest bathing can complement actions developed in Primary Health Care (PHC), such as health promotion groups, corporal practices and mental health care strategies, as well as with Environmental Health Surveillance, highlighting the role of the environment as a protective factor of health. In addition, the practice is supported by PNPIC, expanding the possibilities of comprehensive care within the scope of SUS.

The articulation with the normative frameworks of the PNPS and the National Environment Policy reinforces the need for intersectoral approaches, involving the sectors of health, environment, education, culture, agriculture, and territorial planning. Such articulation is essential to ensure the sustainability of interventions and expand the population’s reach with the strengthening of Conservation Units. By promoting the oriented and educational use of protected natural areas, this practice can contribute to social appreciation of these territories, recognizing them as spaces that promote health, well-being, and environmental education.

The sensory experience in natural environments favors environmental education processes, by stimulating care for nature, the recognition of biodiversity as a common good, and the understanding of ecosystems as determinants of health. In this perspective, forest bathing goes beyond the recreational character, configuring itself as a pedagogical strategy capable of strengthening affective ties with the territory and encouraging conservation practices. When articulated to criteria of planning, participatory management and environmental load capacity, the incorporation of forest bathing in protected areas can generate simultaneous benefits for human health and environmental conservation.

It is also important to consider the cultural appropriation of *Shinrin-Yoku* in Brazilian contexts, respecting local knowledge and community practices historically developed by indigenous peoples, traditional communities, peasants and agroecologists. The incorporation of forest bathing as a health promotion strategy must therefore dialogue with these experiences, valuing the collective, territorial and emancipatory character of care actions and avoiding the uncritical transposition of international models.

## 5 Conclusion

In summary, the findings of this pilot study reinforce that regular and relatively brief contact with nature can produce measurable effects on physiological stress, providing empirical support to the results observed and strengthening the biological plausibility of the benefits associated with interventions based on natural environments. However, there are still gaps regarding the cost-effectiveness of the evaluated intervention and its sustained population impact in the long term.

Future research should consider larger and more heterogeneous samples, test protocols with different intensities and durations of exposure and explore the potential of forest bathing as a complementary strategy to prevent diseases, especially in vulnerable populations, as well as the need to include groups with critical conditions (stage 2 or 3 hypertension, stress, anxiety, and depression) in the variables evaluated to determine the effectiveness of the intervention program. Furthermore, it is necessary to incorporate validated instruments to assess changes in connection with nature, environmental attitudes, and pro-environmental behaviors in both the intervention and control groups, allowing for a more comprehensive evaluation of the broader psychosocial and ecological impacts of forest bathing. In addition, the incorporation of validated instruments to assess connection with nature, environmental attitudes, and pro-environmental behaviors is strongly recommended, enabling a more comprehensive evaluation of the psychosocial and ecological outcomes associated with these interventions.

From a methodological standpoint, future research should adopt more rigorous experimental designs, including stratified randomization procedures to ensure the balanced distribution of important clinical variables (e.g., hypertension status, medication use) among the groups. Improved control and documentation of pharmacological treatments and other health-related variables will minimize potential confounding effects. Furthermore, studies should incorporate systematic tracking of dropout reasons and implement targeted retention strategies, such as enhanced engagement protocols and flexible schedules, to reduce dropout rates and improve adherence.

Also, further refinement of the control groups is needed to ensure greater comparability with the intervention groups in terms of duration, cognitive demand, physical activity, and social interaction. The development of more engaging and structurally equivalent control protocols will help reduce differential dropout and strengthen causal inference. Considering the wide availability of green areas in Brazil and the growing interest in nature-based therapies, it is essential to advance the equitable incorporation of these interventions, ensuring that vulnerable populations have access to sustainable strategies for enhancing well-being, emotional regulation and cardiovascular health. Additionally, integrating reflective guidelines for protocol adaptations and sensitivity to population-specific needs can support scalability and generalizability of future interventions.

Thus, forest bathing emerges as a promising practice for strengthening extended models of health care, contributing to the integration between health and environment and the construction of more sensitive, collective and sustainable responses to contemporary challenges of Public Health.

## Data Availability

The original contributions presented in the study are included in the article/Supplementary material, further inquiries can be directed to the corresponding author.
