# Supplementary material for: Forest bathing (Shinrin-Yoku) as an integrative strategy for mental and cardiovascular health: a quasi-experimental study in Brazil
Source: Front Public Health. 2026 May 11;14:1817791. doi: 10.3389/fpubh.2026.1817791 (PMC13200554; doi:10.3389/fpubh.2026.1817791)
Supplement: Supplementary file 1 [file Table_1.docx]

**Supplementary material**

**Table 1S.** Comparison of the differences between the final and initial values ​​of the continuous variables in the control and intervention groups.

| **Variables** | **Control** | | | | | |  | **Intervention** | | | | | | **p-value*** |
| --- | --- | --- | --- | --- | --- | --- | --- | --- | --- | --- | --- | --- | --- | --- |
|  | **n** | **Mean** | **SD** | **Median** | **Q1** | **Q3** |  | **n** | **Média** | **DP** | **Median** | **Q1** | **Q3** |  |
| PAS (mmHg) | 12 | -9.67 | 6.93 | -10.00 | -14.00 | -4.00 |  | 19 | -15.53 | 17.47 | -9.00 | -22.00 | -5.00 | 0.556 |
| PAD (mmHg) | 12 | -3.17 | 7.33 | -3.00 | -9.00 | 1.50 |  | 19 | -1.68 | 9.57 | 0.00 | -7.00 | 2.00 | 0.569 |
| Cortisol (µg/dL) | 12 | -0.03 | 0.08 | -0.07 | -0.09 | 0.02 |  | 18 | -0.13 | 0.14 | -0.13 | -0.23 | -0.04 | 0.022 |

*Two-sample Wilcoxon rank-sum (Mann-Whitney) test

Elaborated by the authors, 2026.

**Table 2S.** Comparison of pre- and post-intervention values ​​of hemodynamic and biochemical variables in the intervention group.

| **Variables** | **Control** | | | | | | | | | | | |  | | **Intervention** | | | | | | | | | | | **p-value*** | |
| --- | --- | --- | --- | --- | --- | --- | --- | --- | --- | --- | --- | --- | --- | --- | --- | --- | --- | --- | --- | --- | --- | --- | --- | --- | --- | --- | --- |
|  | **n** | | **Mean** | | **SD** | | **Median** | | **Q1** | | **Q3** | |  | | **n** | | **Média** | | **DP** | | **Median** | | **Q1** | | **Q3** |  |  |
| PAS (mmHg) | 20 | 125.70 | | 19.11 | | 119.50 | | 112.00 | | 131.50 | |  | | 21 | | 108.95 | | 10.22 | | 108.00 | | 102.00 | | 114.00 | | | 0.001 |
| PAD (mmHg) | 20 | 73.20 | | 8.02 | | 74.00 | | 69.50 | | 77.50 | |  | | 21 | | 70.95 | | 10.32 | | 70.00 | | 64.00 | | 76.00 | | | 0.504 |
| Cortisol (µg/dL) | 22 | 0.31 | | 0.10 | | 0.29 | | 0.24 | | 0.37 | |  | | 18 | | 0.17 | | 0.07 | | 0.16 | | 0.11 | | 0.21 | | | 0.002 |

*Wilcoxon signed-rank test

Elaborated by the authors, 2026.

**Table 3S.** Comparison of the differences between the final and initial scores of the PSS-10 and DASS-21 in the control and intervention groups.

| **Variables** | **Control** | | | | | | | | | | | | |  | | **Intervention** | | | | | | | | | | | **p-value*** | |
| --- | --- | --- | --- | --- | --- | --- | --- | --- | --- | --- | --- | --- | --- | --- | --- | --- | --- | --- | --- | --- | --- | --- | --- | --- | --- | --- | --- | --- |
|  | **n** | | **Mean** | | **SD** | | **Median** | | **Q1** | | | **Q3** | |  | | **n** | | **Média** | | **DP** | | **Median** | | **Q1** | | **Q3** |  |  |
| PSS-10 | | 12 | | -2.08 | | 2.23 | | -2.50 | | -4.00 | 0.00 | |  | | 23 | | -5.30 | | 5.91 | | -5.00 | | -10.00 | | -2.00 | | | 0.046 |
| DASS depressão | | 12 | | -1.17 | | 4.78 | | 0.00 | | -2.00 | 1.00 | |  | | 23 | | -4.61 | | 9.35 | | -2.00 | | -10.00 | | 0.00 | | | 0.378 |
| DASS ansiedade | | 12 | | -1.50 | | 2.84 | | 0.00 | | -4.00 | 0.00 | |  | | 23 | | -6.09 | | 8.03 | | -4.00 | | -10.00 | | -2.00 | | | 0.040 |
| DASS estresse | | 12 | | -2.00 | | 2.83 | | -1.00 | | -5.00 | 0.00 | |  | | 23 | | -7.48 | | 9.71 | | -4.00 | | -16.00 | | 0.00 | | | 0.130 |

*Two-sample Wilcoxon rank-sum (Mann-Whitney) test

Elaborated by the authors, 2026.
